# Supplementary material for: Histological Characterization of Class I HLA Molecules in Whole Umbilical Cord Tissue Towards an Inexhaustible Graft Alternative for Reconstructive Surgery
Source: Bioengineering (Basel). 2023 Jan 12;10(1):110. doi: 10.3390/bioengineering10010110 (PMC9855378; doi:10.3390/bioengineering10010110)
Supplement: Supplementary file 1 [file bioengineering-10-00110-s001.zip › bioengineering-2145162-supplementary.pdf]

Supplementary Figures and Tables

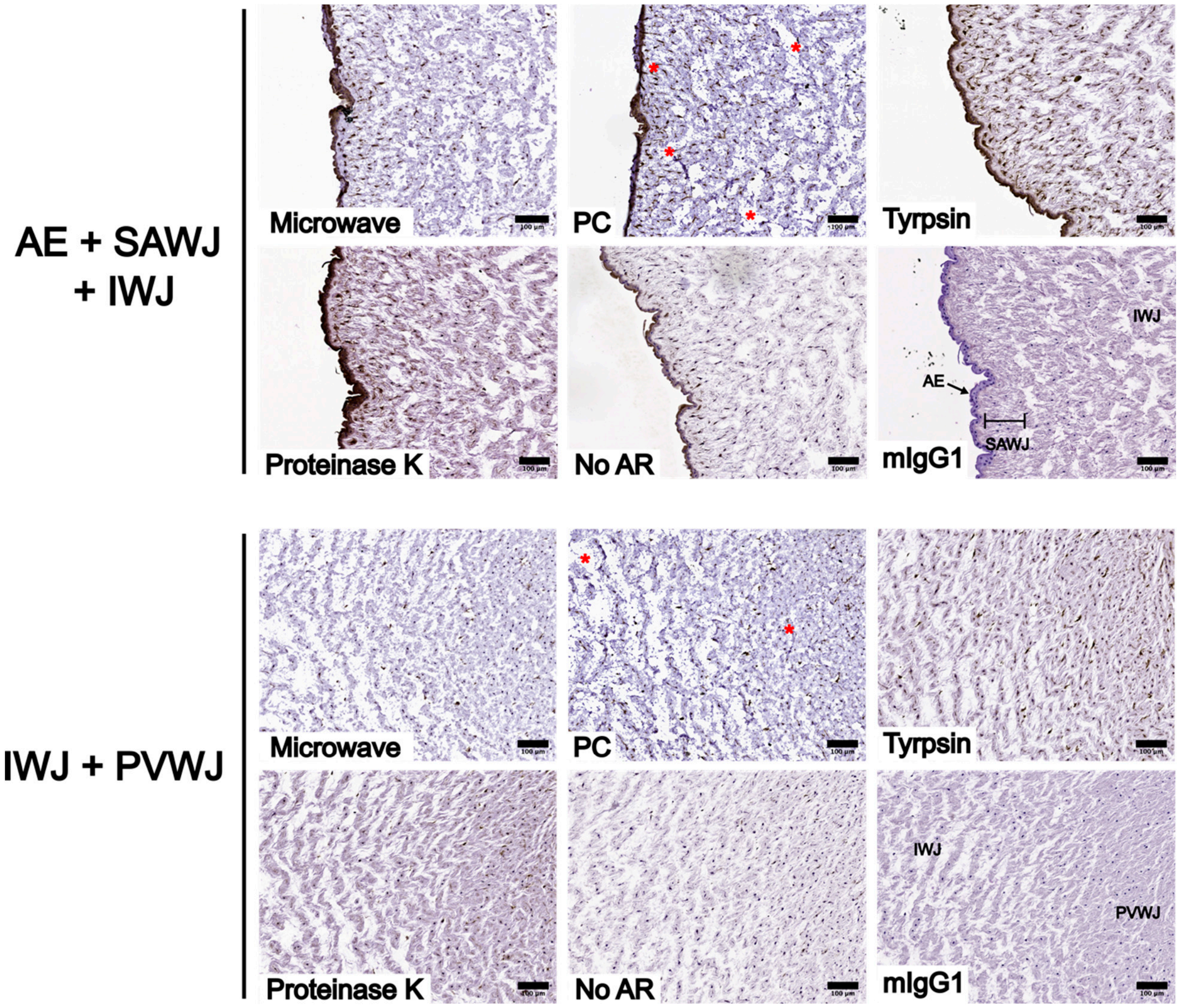

Figure S1. Comparison of HLA-ABC IHC staining results in UC tissue cross-sections at term using five different antigen retrieval protocols for IHC protocol optimization with a focus on the AE and WJ subregions. **Top panel:** the amniotic epithelium (AE), subamniotic Wharton's Jelly (SAWJ), and intermediate Wharton's Jelly (IWJ) regions of UC tissue cross-sections after each antigen retrieval (AR) protocol. **Bottom panel:** the IWJ and perivascular Wharton's Jelly (PVWJ) subregions of UC tissue cross-sections at term after each AR protocol. Red asterisks: examples of areas in pressure cooker (PC) treated-tissue sections where the tissue damage is more severe than microwave-treated tissue sections. The corresponding AR protocol and isotype control used are indicated at the bottom left of each tissue section image. Scale bars=100 μm. Abbreviation: anti-mouse IgG1 antibody (mIgG1).

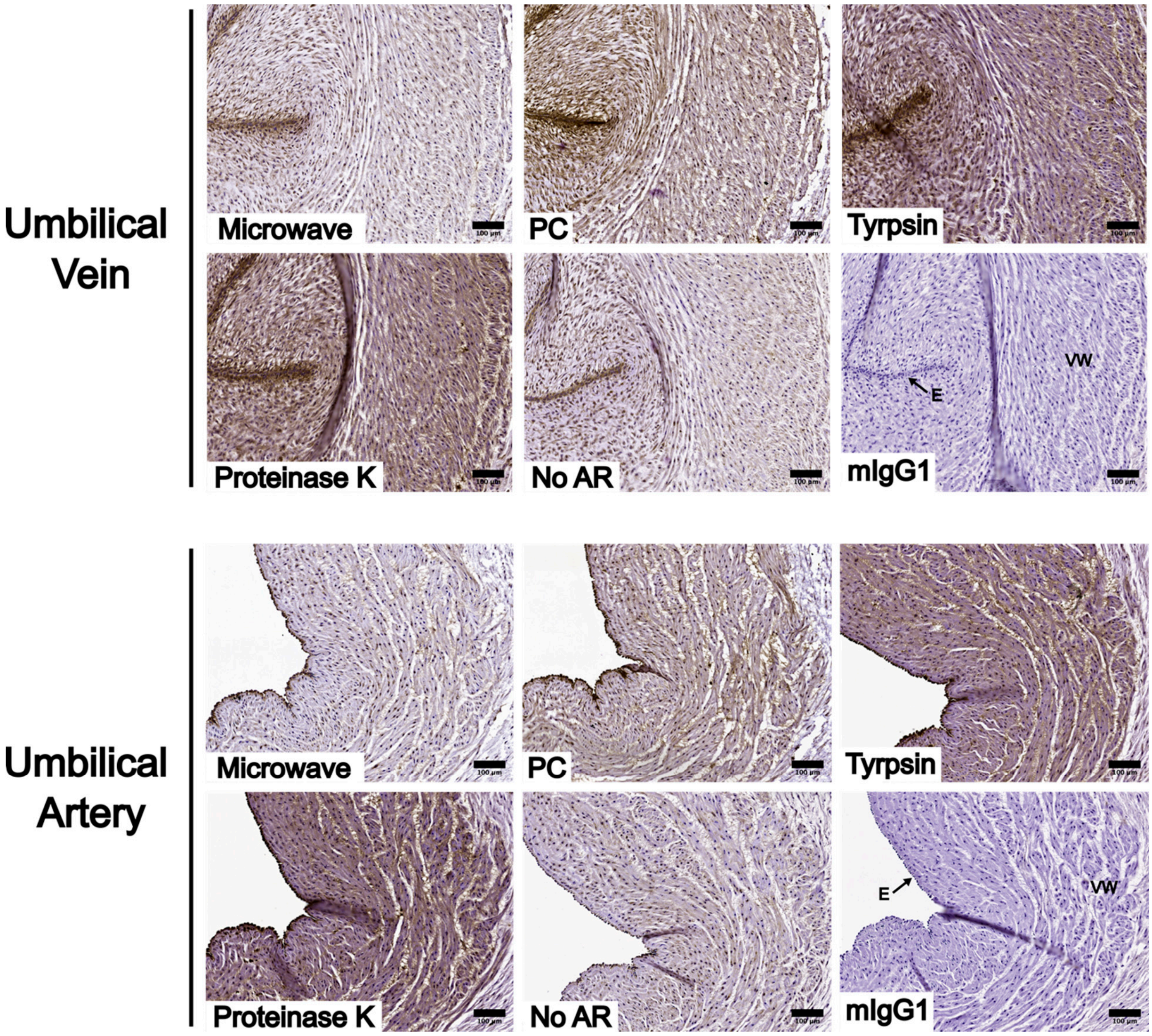

Figure S2. Comparison of HLA-ABC IHC staining results in UC tissue cross-sections at term using five different antigen retrieval (AR) protocols for IHC protocol optimization with a focus on umbilical vessels. **Top panel:** one region of the umbilical vein of the stained UC tissue sections. **Bottom panel:** one region of the umbilical artery of the stained UC tissue sections. The corresponding AR protocol and isotype control used are indicated at the bottom left of each tissue section image. Scale bars=100 μm. Abbreviations: endothelium(E); pressure cooker (PC); vessel wall (VW).

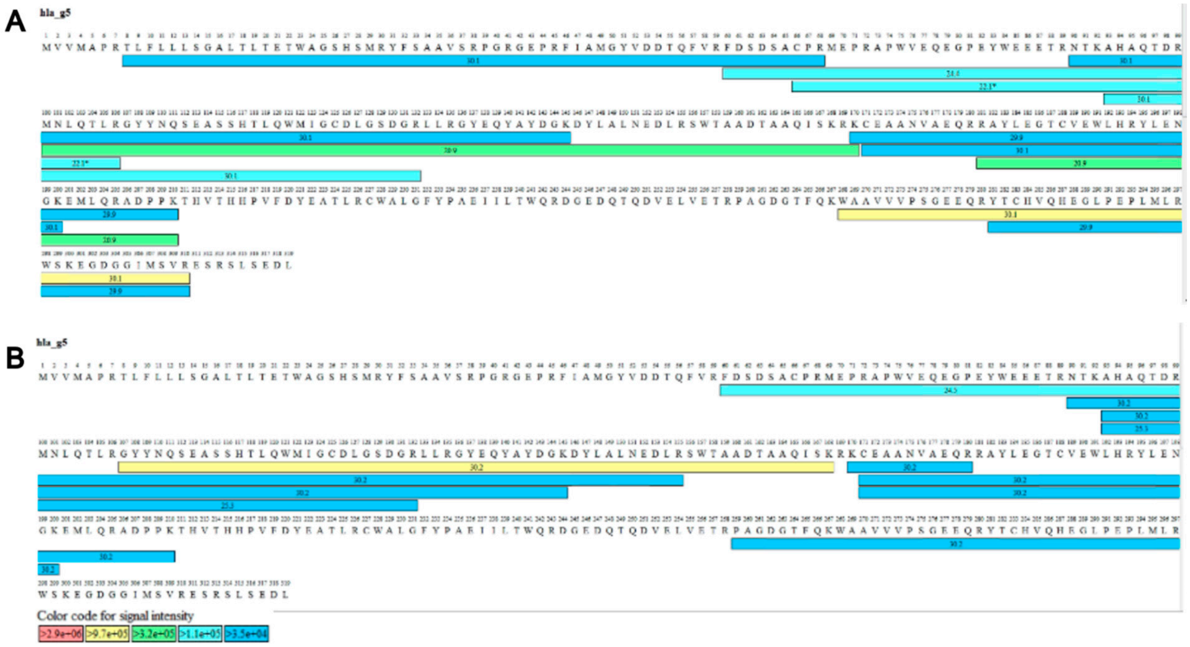

Figure S3. Peptide alignment maps from the mass spectrometry analysis of whole UC tissue at term. **(A)** Peptide alignment map of the UC tissue lysate sample. **(B)** Peptide alignment map of the negative control.

Table S1. Comparison of HLA-ABC IHC staining results using three different primary antibody working concentrations with five different AR protocols in term placenta

**AR**	**1° Antibody Working Concentrations (μg/mL)**		
	0.25	0.125	0.05
Microwave	++	+	+# (Present)
PC	++ (Present)	++# (Present)	+#
Trypsin	++#	++	+ (Present)
Proteinase K	+++#	++# (Present)	++(Present)
No AR	+ (Present)	0# (Present)	0# (Present)

Data is presented as staining intensity score (presence or absence of any tissue disruption).
Presence of non-specific binding in isotype control. Staining intensities were graded as such: 0 (no staining or distinct differences from the isotype control), + (weak brown staining), ++ (moderate brown staining), +++ (strong dark brown staining).

Table S2. Summary of class I HLA IHC staining intensities in UC tissue cross-sections at term (n=10).

**UC region**	**Intensity Score**	**Number of samples showing positive HLA staining**		
		HLA-ABC	HLA-E	HLA-G
AE	0	0	1	10
	+	0	7	0
	++	8	2	0
	+++	2	0	0
WJ	0	0	1	10
	+	1	7	0
	++	9	2	0
	+++	0	0	0
Endothelium	0	0	0	10
	+	0	1	0
	++	6	7	0
	+++	4	3	0
VW	0	0	1	10
	+	5	9	0
	++	5	0	0
	+++	0	0	0

Table S3. Summary of class I HLA IHC staining intensities in UC tissue cross-sections at 12-19 weeks (n=10).

**UC region**	**Intensity Score**	**Number of samples showing positive HLA staining**		
		HLA-ABC	HLA-E	HLA-G
AE	0	0	2	10
	+	0	6	0
	++	7	2	0
	+++	3	0	0
WJ	0	0	2	10
	+	4	6	0
	++	4	2	0
	+++	2	0	0
Endothelium	0	0	1	10
	+	1	2	0
	++	6	6	0
	+++	3	1	0
VW	0	0	4	10
	+	2	6	0
	++	7	0	0
	+++	1	0	0

Table S4. Summary of HLA-ABC and HLA-E presence in WJ subregions of UC tissues at term and 12-19 weeks (n=10 each).

**Gestational Age**	**HLA**	**SAWJ***	**IWJ***	**PVWJ***
Term	HLA-ABC	10	10	10
	HLA-E	9	8	9
12-19 weeks	HLA-ABC	10	10	10
	HLA-E	5	7	8

*The number of UC samples that contained HLA^+ cells.
